# Chronic hepatitis C: Diagnosis and treatment made easy

**DOI:** 10.1080/13814788.2022.2056161

**Published:** 2022-05-17

**Authors:** Naim Abu-Freha, Binil Mathew Jacob, Ali Elhoashla, Zaid Afawi, Talab Abu-Hammad, Foad Elsana, Sergey Paz, Ohad Etzion

**Affiliations:** a The Institute of Gastroenterology and Hepatology, Soroka University Medical Center and the Faculty of Health Sciences, Ben-Gurion University of the Negev, Beer-Sheva, Israel; b Medical School for International Health, Ben-Gurion University of the Negev, Beer-Sheva, Israel; c Clalit Health Services, Beer-Sheva, Israel; d Erasmus MC, Rotterdam, The Netherlands

**Keywords:** Hepatitis C virus, direct antiviral agents, family physicians

## Abstract

**Background:**

Hepatitis C Virus (HCV) is a common cause of chronic liver disease and its ensuing complications. In the last years, there has been a revolution of the treatment for patients with HCV regarding efficacy, simplicity, safety and duration of treatment. The role of the family physician is vital in all steps of care: screening, diagnosis, linkage to treatment, treatment and follow-up.

**Objectives:**

This review aims to summarise the family physician and the important updated recommendations for diagnosis and treatment of patients with chronic HCV.

**Methods:**

The updated recommendations were reviewed and summarised in a short and simple review.

**Results:**

Patients with any risk factor for HCV should first be screened for HCV antibodies. In the case of positive antibodies, reflex testing for RNA polymerase chain reaction (PCR) should be done without waiting for genotype. For patients with positive PCR, fibrosis assessment should be conducted using laboratory panels (Fibrosis-4 index (FIB-4) or aspartate aminotransferase to platelet ratio index (APRI)); if advanced fibrosis is suspected, additional non-invasive fibrosis assessment is needed, such as fibrotest or liver elastography. Naïve non-cirrhotic or compensated cirrhosis (Child-Pugh-Score A) could be treated with pangenotypic drugs, Glecaprevir/pibrentasvir (Maviret) for eight weeks, or Sofosbuvir/velpatasvir (Epclusa) for 12 weeks.

**Conclusion:**

Patients without advanced fibrosis and comorbidities can be treated by the educated family physician. However, patients with comorbidities, cirrhosis or coinfection (HIV, Hepatitis B Virus (HBV)) should be referred to the liver clinic. In case of screening patients with risk factors or likelihood of dormant HCV, health organisations should provide the appropriate resources, logistics, finances and workforce.

Key messages

- Family physicians have a central role during screening, diagnosis, linkage to treatment, treatment and follow up of chronic hepatitis C (HCV) patients.
- Chronic HCV treatment is simple, using a direct-acting antiviral with high efficacy.
- Naïve, non-cirrhotic without comorbidities patients can be treated by the educated family physician.

## Introduction

Hepatitis C Virus (HCV) is a leading cause of chronic liver disease, with an estimated 71 million people worldwide infected with the virus [1], this equates to an estimated prevalence of 0.64% of the total population of the European Union and an estimated prevalence of 2–5% in the Eastern European countries [2–4].

HCV is a positive-sense, single-stranded RNA flavivirus with seven known subtypes defined according to the different genotypes, 1a, 1 b, 2, 3, 4 and 6. These genotypes have varying prevalence across countries. Due to HCV’s propensity for chronic necroinflammation, it remains a leading cause for chronic hepatopathies including liver cirrhosis and hepatocellular carcinoma [1]. In addition, employees infected with HCV had lost significantly more workdays, including sick leave, short-term disability and long-term disability [5].

Treatment of HCV is unparalleled among viral therapeutics and has thus been considered a medical revolution, especially with the switch to oral therapy showing little to no side effects, high effectiveness and relatively short time needed for definitive treatment.

In the era of simplified, direct-acting antiviral treatment with high success rate, one of the most important steps in diagnosing and treating patients with chronic hepatitis C is to discover the undiagnosed patients and treat them in the community or at liver clinics. This critical step cannot be implemented without the cooperation and action of primary care practitioners. This review summarises the important, most updated information in terms of screening, diagnosis and treatment of patients with chronic HCV discussing the important role of the family physician, especially the challenges they need to overcome.

### Transmission and risk factors

HCV is transmitted by blood or body fluids. The most common modes of transmission are through sharing needles among drug users and any blood transfusion before 1992, the year that standardised testing was implemented on blood products [6]. Additional risk factors for transmission include infants born to HCV viraemic mothers, haemodialysis, HIV positivity, sexual intercourse of men who have sex with men, organ transplant before 1992, people migrated from a country with high prevalence and reception of clotting factor concentrate before 1987 [6,7].

### Natural history and clinical consequences

Chronic infections with HCV could result in chronic inflammation of the liver tissue. This liver injury ranges from minimal necro-inflammation to cirrhosis, as well as late complications of cirrhosis such as hepatocellular carcinoma (HCC). Treatment of HCV reduces long-term complications, cirrhosis, HCC and all-cause mortality [8–15].

## HCV and ethnic minorities

The prevalence of HCV among specific ethnic minorities varies both across European countries as well as within them. For example, in England, the whole population prevalence of HCV was estimated at 0.67% but a different prevalence was found among people who migrated from south Asian countries: 1.9% among people of Pakistani origin, 0.32% among those of Bangladeshi origin and 0.27% for those of Indian origin [16]. A higher HCV prevalence is expected for individuals who migrated to Europe from countries with high HCV prevalence [17]. Additional studies found that ethnic minority populations are more likely to have higher morbidity and mortality due to chronic hepatitis C, the reasoning for which could be related to barriers accessing health services, longer duration of undetected or untreated infection and prevalence of comorbidities [17]. The local data regarding risk groups, specifically from the lens of ethnic minorities, are important to the family physician in every country for the dual objectives of improved screening on the one hand, and on the other, for promoting awareness and access to treatment.

### Diagnosis

Most patients with acute HCV infection will develop chronic infection (about 85%), whereas the virus will be cleared spontaneously in about 15% of this population. Patients infected with HCV will have positive HCV antibodies but only the positive viral load (PCR) confirms active infection. Since these antibodies persist, patients can test positive for cases of past infection (spontaneous clearances), previously treated infection or false-positive result. Therefore, the presence of active HCV infection, or relapse of underlying infection, can only be confirmed by positive RNA PCR. All patients suspected of HCV infection should be tested for antibodies and if the antibodies are positive, further testing for HCV RNA is warranted. Patients with positive antibodies but negative RNA PCR should be informed that they are not protected from reinfection although they do not have active infection. Genotype testing in the era of pan-genotypic treatment is no longer needed by most recommendations.

### HCV RNA reflex testing

Reflex testing for HCV RNA (using additional blood sample) is an alternative option to RNA PCR for cases of positive antibodies, both recommended and accessible at most laboratories. Reflex testing is critical to shortening the preparatory phase of HCV treatment and improving the linkage for care. This methodology is helpful to the hepatologist/gastroenterologist who can thereby make an informed decision regarding the type of therapeutic as early as the first meeting. RNA PCR reflex testing is recommended by the European Association for the Study of the Liver (EASL) and the American Association for the Study of the Liver Disease (AASLD) [18,19].

### Screening

Since a significant portion of patients infected with HCV is unaware of their infection status, it is paramount for any patient with known risk factors to be screened. Active intervention is necessary for HCV antibody screening in those patients with high risk for HCV; particularly screening is critical for high-risk populations such as intravenous drug users or individuals with limited access to the health system. Additional special considerations should be made for specific, marginalised groups such as but not limited to the prison population, refugees, ethnic minorities and people of lower socioeconomic status. In these cases, the use of the rapid diagnostic tests (RDTs) and dry blood spot with high sensitivity could be helpful [20,21].

### Assessment of liver fibrosis

Invasive liver biopsy used to be the best option for assessing liver fibrosis in the past; however, nowadays imaging and labs panel testing are available, namely fibroscan and fibrotest. Fibroscan (liver elastography) or fibrotest (calculation of Alpha-2-macroglobulin, Haptoglobin, Apolipoprotein A1, Gamma-glutamyl transpeptidase (GGT), Total bilirubin, and Alanine transaminase) have essentially replaced the liver biopsy for fibrosis assessment testing in chronic HCV patients. Nowadays, biomarkers for fibrosis are recommended, since this testing is simple, inexpensive and readily available. Most common fibrosis panels are FIB-4 and APRI (AST to Platelet Ration Index), which involve calculations of simple, readily available blood tests. FIB-4 test includes calculation of age, AST, ALT and platelets, whereas APRI is an AST to platelets ration index [18]. In case of suspicion of advanced fibrosis, an additional fibrosis assessment is needed (Fibrotest/Fibroscan). FIB-4 < 1.45 has a negative predictive value of 90% for advanced fibrosis [22]. Several scores are used for the histological fibrosis assessment, of which the non-invasive modalities grading scores are comparable with the Metavir score, which is used as scoring for liver biopsy. Five different grades are used, F0 for no fibrosis, F1 for mild fibrosis (fibrous portal expansion), F2 for moderate fibrosis (periportal septae), F3 for severe fibrosis (portal-central septae) and F4 for cirrhosis [23]. Patients with asymptomatic cirrhosis are referred to as having compensated cirrhosis, whereas patients considered to have decompensated cirrhosis are those with development of complications from portal hypertension or liver dysfunction such as ascites, varices bleeding, liver failure, hepato-renal syndrome or encephalopathy.

### Additional assessment before treatment

Additional evaluations are needed before treatment of HCV, including laboratories, imaging and recommendation for vaccinations. The important pre-treatment laboratories and further recommendations are summarised in Table 1.

**Table 1.** Pre-treatment requirements for patients with chronic HCV.

Pre-treatment	
Complete blood count	
Biochemistry testing	Liver enzymes, kidney function, electrolytes, albumin
Coagulation function	INR – international normalised ration
Hepatitis B serology for current or past infection	HBs antigen, anti-HBc antibodies and anti-HBs antibodies
Hepatitis A Virus (HAV)	HAV antibodies
HIV	HIV antibodies
HBV and HAV vaccination	For patients who are not immune
Preparation for treatment	
HCV antibodies	
HCV RNA	
Fib 4 calculation	If needed, fibrosis assessment (fibrotest or fibroscan)
Liver ultrasound	

### Towards ending viral hepatitis – a World Health Organization (WHO) initiative

The WHO’s initiative for eliminating HCV was proposed in 2016, with the ambition of targeting 90% reduction in chronic HCV incidence by the year 2030 as well as 65% reduction in mortality, compared with 2015 levels [24]. The proposed strategy includes both reductions in incidence and mortality on the one hand and on the other hand, increased treatment accessibility for all patients. This WHO initiative has been adopted, implemented and monitored at the national level by a diverse set of member states.

### Treatment of HCV

The European Association for the Study of the Liver (EASL) [18], the American Association for the Study of the Liver Disease (AASLD) and local national associations updated their recommendations regarding the treatment of HCV in the past few years [19]. The updated recommendations focus on simplifying the diagnosis, pre-treatment requirements and treatment. The rate of treatment success is very high, reaching about 95–100% in several studies [25]. The success rate depends on the genotype, fibrosis grade and whether the patient is naïve or experienced.

Most common drug therapeutics approved for hepatitis C will be shortly summarised in this review.

### Sofosbuvir/velpatasvir (Epclusa, pan-genotypic)

A combination of 400 mg sofosbuvir and 100 mg velpatasvir in a single tablet is taken orally once daily, with or without food. It is used as a pan-genotypic drug for treatment of all HCV genotypes. The treatment duration is 12 weeks among both naïve or experienced patients and among compensated cirrhotic patients.

### Glecaprevir/pibrentasvir (Maviret, pan-genotypic)

Glecaprevir and pibrentasvir are used as a pan-genotypic treatment for HCV and contain 100 mg glecaprevir and 40 mg pibrentasvir, to be taken as three tablets, once daily with food. The treatment duration is eight weeks for naïve non-cirrhotic or compensated, cirrhotic patients; however, 12 weeks treatment is recommended for experienced patients with compensated cirrhosis (Child-Pugh A). Special consideration is essential for genotype 3, whereas 8 weeks treatment for naïve non-cirrhotic patients and 12 weeks for experienced non-cirrhotic patients is recommended, patients with genotype 3 and compensated cirrhosis should be treated for 8–12, whereas for compensated experienced patients with cirrhosis, 16 weeks are recommended [18].

### Grazoprevir/elbasvir (Zepatier, genotype 1 b)

Containing 100 mg grazoprevir and 50 mg elbasvir in a single tablet, dosage is one tablet once daily, with or without food. It is indicated for patient with known 1 b genotype of 1 b, given good previous experience.

### Sofosbuvir/velpatasvir/voxilaprevir (after DAA failure, pan-genotypic)

A combination of 400 mg sofosbuvir, 100 mg velpatasvir and 100 mg voxilaprevir, taken as one tablet, is used as pan-genotypic treatment for patients who have experienced treatment failure with direct-acting antiviral therapy (DAA).

### Diagnosis and treatment strategy

**Figure 1.**
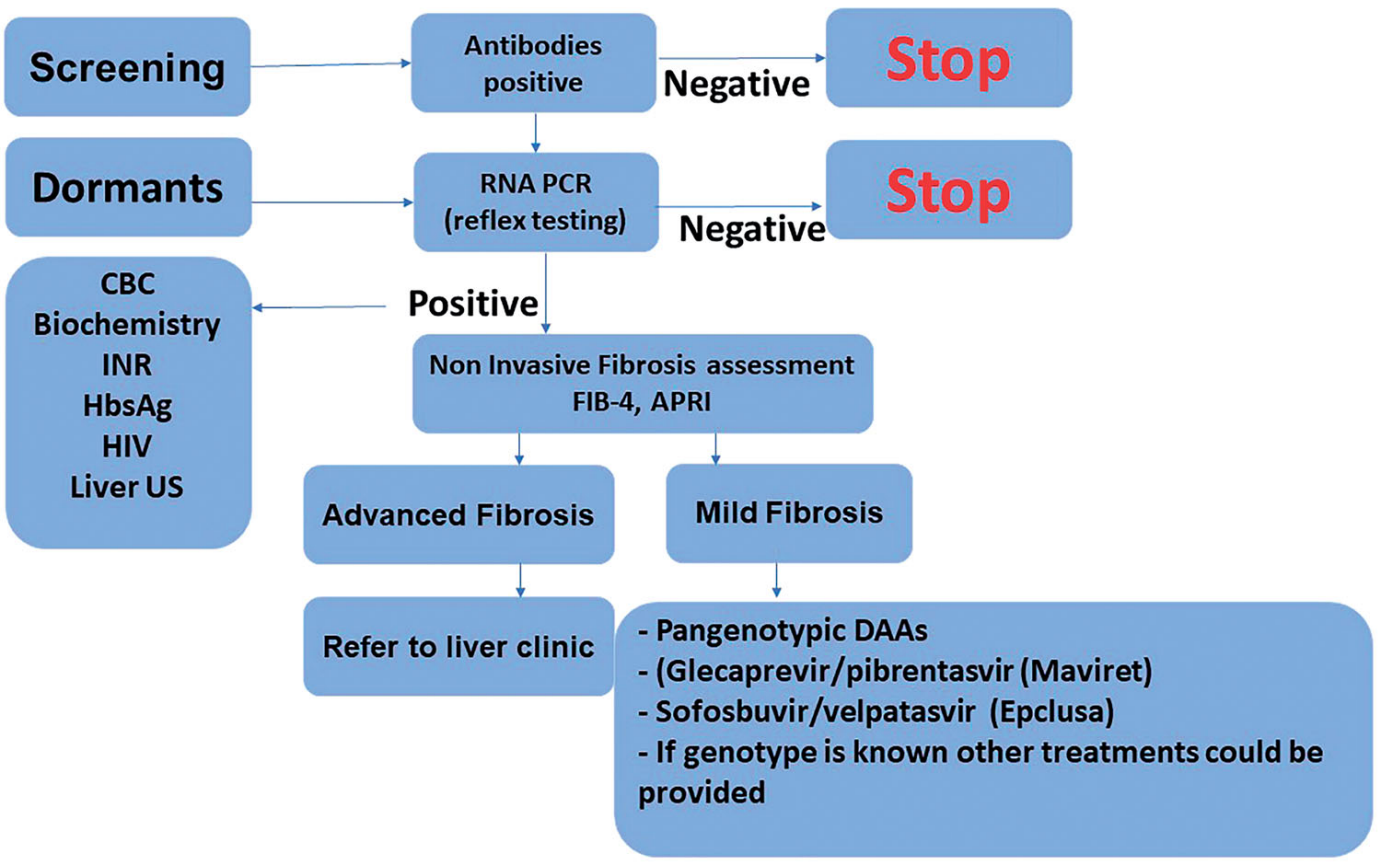
Screening, diagnosis and treatment of chronic hepatitis C.

In the highly effective direct-acting antivirals (DAA) treatment era, the diagnosis and treatment process has become short and simple. Although in the past, various pre-treatment requirements were needed, followed by several visits to the hepatologist or gastroenterologist to establish definitive diagnosis, today the decision-making process has become less laborious. The important parts of the process include screening and linkage to treatment, meaning any patient with risk factors for HCV should be screened; if HCV antibodies are positive, then reflex testing for HCV RNA should be performed. Genotype testing is no longer needed in most countries due to the availability of pan-genotypic treatment. In most individuals, the Fib-4 calculator or APRI index can be used for fibrosis assessment and only among patients suspected to have advanced fibrosis or high Fib-4 score would any additional fibrosis assessment (fibroscan or fibrotest) be performed. After treatment with DAAs, additional HCV RNA test for 12 weeks after treatment is needed to confirm sustained viral response (SVR). Patients with advanced fibrosis, relapses or comorbidities should be treated and followed in a liver clinic. In cases of cirrhosis, hepatic carcinoma screening is required. Screening, diagnosis and treatment of HCV made easy and summarized in Figure 1.

### Monitoring treatment

In the past, several RNA PCR tests were needed; however, current guidelines necessitate only two tests: once before treatment (during 6 months) and once 12 weeks post treatment to ensure Sustained Virologic Response (SVR). For patients with mild fibrosis F0-2, no specific additional follow-up is needed after SVR but patients with advanced fibrosis F3-4 should be monitored by the Hepatologist/Gastroenterologist, and Hepatocellular carcinoma screening is recommended.

### Side effects

Side effects in patients treated with DAA are relatively common but mild. Headache, nausea, fatigue and diarrhoea are most frequent, whereas more severe side effects are uncommon [25].

### Drug–drug interactions

Drug–drug interaction is an important issue among a number of HCV patients, primarily because these patients are young individuals. Drug–drug interaction should be considered in those patients undergoing chronic treatment for any chronic disease. Important drug groups that may interact with DDAs are antiretroviral treatments, lipid-lowering treatments, antidepressant and antipsychotics, cardiovascular, antiplatelets, anticoagulation and anticonvulsants. Detailed information about interactions of DDAs and the groups as mentioned above are available in the EASL and AASLD guidelines [18,19] and the online resource maintained by the University of Liverpool, https://www.hep-druginteractions.org.

### Role of the family physician

In most countries, the family physician plays a key role in the different steps of the process: screening, linkage to treatment and treatment of patients with chronic HCV. Every family physician should familiarise themselves with the common risk factors for HCV in their region and ensure such patients in their care are adequately screened for HCV. In cases of positive antibodies, the following steps related to the preparation for treatment should be conducted by the family physician. In countries where family physicians have undergone the appropriate education they may treat patients with mild fibrosis (according to the local/national recommendations for treatment) but patients with advanced fibrosis should be treated in the liver clinic.

### Challenges to resolve

Challenges related to screening, diagnosis and treatment should be resolved in every country, especially when it comes to actionable change at the level of local health system facilities. Cooperation by the family physician in this process is instrumental since they have the medical knowledge and united advocacy to support patients’ getting optimal treatment. Patients with any risk factor for chronic HCV should be screened for HCV antibodies by the family physician, followed by the subsequent reflex testing for HCV RNA. ‘Dormant’ patients are patients with previous positive HCV AB and RNA but were never treated. This group of patients is an important group for whom intervention should be actively sought after; patients should be invited for updated labs and treated. Another critical challenge is out-of-pocket costs and insurance coverage. If treatment is not made financially available, only a portion of this patient group will be treated. These and similar challenges regarding the screening, diagnosis, and treatment should be resolved in every health system with the cooperation of the relevant health maintenance organisations.

## Discussion

Family physicians must be familiar with the updated strategy and recommendations regarding chronic hepatitis C screening, diagnosis and treatment. Several barriers in terms of diagnosis and treatment process were overcome in many countries, which should now be adopted; reflex RNA testing, genotype and fibrosis assessment can no longer be factors delaying the progress to treatment.

International and national associations have published updated clinical practice; however, these publications are too comprehensive, more so appropriate for specialists than for primary care physicians [18,19]. In this short review, the important issues regarding chronic hepatitis C were summarised to help family physicians be well informed and involved in the treatment of chronic hepatitis C. Furthermore, family physicians need to familiarise themselves with this short list of medications as treatment of HCV is no longer extremely complex since DAAs are highly effective and oral treatments are without side effects.

On the one hand, the family physician should be aware of the important and most common risk factors for HCV in their region and actively screen their patient for HCV antibodies; on the other hand, the health maintenance organisations also have an important role to play in organising the different parts for screening, linking to treatment and follow-up. A detailed plan should be carried out, including retrieving data from the computerised system regarding individuals with known risk factors for HCV or known positive HCV antibodies or RNA. The process of screening and linking to treatment should be short and simple. The family physician should take care of all treatment preparations so when consulting the hepatologist/gastroenterologist, an appropriate treatment can be prescribed. In countries, where family physicians are educated in chronic HCV treatment, they can treat patients with mild fibrosis and refer patients with advanced fibrosis to liver clinic. Patients with chronic HCV infection and decompensated cirrhosis, hepatocellular carcinoma or comorbidities such as solid organ transplant recipients, cryoglobulinemia or lymphoma, chronic kidney failure, coinfection with HIV or hepatitis B should be referred to the liver clinic [18,19]. The primary care physician could treat naïve patients with chronic HCV and without comorbidities or advanced fibrosis using a simplified protocol; glecaprevir/pibrentasvir (Maviret) for 8 weeks or sofosbuvir/velpatasvir (Epclusa) for 12 weeks [18,19]. Human maintenance organisations have to allocate the appropriate resources, logistics, finances and workforce. Special consideration is needed for specific socially marginalised groups, such as intravenous drug users and patients in prison, for whom appropriate services should be prepared.

## Conclusion

Screening, linkage to treatment and treatment of patients with chronic hepatitis C are important in the critical process of eliminating HCV. Family physicians should be up-to-date regarding all steps of diagnosis and treatment of HCV and can choose to be involved and have a central role in the treatment of patients with mild fibrosis.
